# 术前恶性不除外手术病理确诊肺部良性病变297例患者临床-放射-病理特征分析：一项中国单中心回顾性队列研究

**DOI:** 10.3779/j.issn.1009-3419.2020.104.24

**Published:** 2020-09-20

**Authors:** 永健 留, 闽江 陈, 超 郭, 巍 钟, 秋月 叶, 静 赵, 晴 周, 晓星 高, 潇衍 刘, 红格 梁, 岳泉 石, 德利娜 蒋, 洪生 刘, 燕 徐, 单青 李, 孟昭 王

**Affiliations:** 1 100730 北京，中国医学科学院，北京协和医学院，北京协和医院呼吸与危重症医学科 Department of Respiratory and Critical Care Medicine, Peking Union Medical College Hospital, Chinese Academy of Medical Sciences and Peking Union Medical College, Beijing 100730, China; 2 100730 北京，中国医学科学院，北京协和医学院，北京协和医院胸外科 Department of Thoracic Surgery, Peking Union Medical College Hospital, Chinese Academy of Medical Sciences and Peking Union Medical College, Beijing 100730, China

**Keywords:** 肺部良性病变, 肺结节, 肺肿瘤, 肺癌筛查, Pulmonary benign lesions, Pulmonary nodule, Lung neoplasms, Lung cancer screening

## Abstract

**背景与目的:**

随着癌症早筛意识的提高，低剂量计算机断层扫描(low-dose computed tomography, LDCT)用于肺癌筛查在中国广泛开展。尽管有部分胸部LDCT筛查所见的肺部病灶是肿瘤病灶，但大多数的肺部结节是良性病变。如何有效的对肺部病灶进行术前鉴别，如何降低部分可避免手术的良性疾病的手术切除比例，是需要关注的问题。

**方法:**

本研究纳入2017年1月1日-2018年12月31日期间北京协和医院诊治的，术前考虑肺部恶性病变不能除外，经手术病理确认为良性病变的患者，回顾性分析患者临床信息。

**结果:**

297例患者纳入本研究，占我院肺部病灶行肺部手术治疗患者的9.8%。197例(66.3%)患者因体检行LDCT筛查发现肺部病灶。肺部病变胸部CT影像学评估情况，可评估的323个病灶，平均长径为(17.9±12.1)mm，直径≥8 mm的占91.0%，实性最多见(212/323, 65.6%)，此类肺部病灶可有毛刺征(71/323, 22.0%)、分叶征(94/323, 29.1%)、胸膜牵拉征(81/323, 25.1%)、血管集束征(130/323, 40.2%)、空泡征(23/323, 7.1%)等，提示恶性病变的影像学特征。292例(98.3%)行电视辅助胸腔镜手术(video-assisted thoracoscopic surgery, VATS)，232例(78.1%)患者行肺楔形切除术，13例(4.4%)行肺段切除术，51例(17.2%)患者行肺叶切除术。4例(1.3%)患者出现手术并发症。术后病理类型前3位的是感染性疾病98例(33.0%)、炎性结节96例(32.3%)和错构瘤64例(21.5%)。

**结论:**

因术前不能排除恶性而行手术切除的肺部良性病灶，影像学表现以实性病灶多见，但多具有提示恶性的影像学特征。VATS可作为一种明确病原病理的重要活检方式。此类病灶病理结果以感染性疾病和炎性结节最为常见，错构瘤第三。

## Abstract (en)

**Background and objective:**

Low dose computed tomography (LDCT) for lung cancer screening is widely employed in China as a result of increasing cancer screening awareness. Although some pulmonary lesions detected by LDCT are cancerous, most of the pulmonary nodules are benign. It is important to make effective preoperative differentiation of pulmonary lesions and to obviate the need for surgery in some patients with benign disease.

**Methods:**

From January 1, 2017 to December 31, 2018, patients in our institution with surgical pathology confirmed benign pulmonary lesions in which malignancy could not be excluded in preoperative assessment were enrolled in this study. Retrospective analysis of clinical data was conducted.

**Results:**

297 cases were collected in this study. Prevalence of benign disease in patients underwent resection for focal pulmonary lesions is 9.8% in our institution. In 197 patients (66.3%), pulmonary lesions were detected by LDCT screening. A total of 323 assessable pulmonary lesions were detected by chest CT. The average diameter of pulmonary lesions was (17.9±12.1) mm, and 91.0% of which were greater than or equal to 8 mm. Solid nodules accounted for 65.6% of these lesions. Imaging characteristics suggesting malignancy were common, including spicule sign (71/323, 22.0%), lobulation (94/323, 29.1%), pleural indentation (81/323, 25.1%), vascular convergence sign (130/323, 40.2%) and vacuole sign (23/323, 7.1%). 292 patients (98.3%) underwent video-assisted thoracoscopic surgery (VATS). Pulmonary wedge resection was performed in 232 cases (78.1%), segmental resection in 13 cases (4.4%) and lobotomy in 51 cases (17.2%). Surgical complications occurred in 4 patients (1.3%). The most frequent findings on surgical pathology analysis were: infectious lesions in 98 cases (33.0%), inflammatory nodules in 96 cases (32.3%), and hamartoma in 64 cases (21.5%).

**Conclusion:**

Solid nodules accounted for most of these benign pulmonary lesions in which malignancy could not be excluded preoperatively, and imaging characteristics suggesting malignancy were common. VATS is an important biopsy method to identify etiology and pathology for lesions. The most frequent benign pulmonary diseases that are suspected to be malignant and underwent surgical resection are: infectious lesions, inflammatory nodules and hamartoma.

肺癌是全球死亡率最高的恶性肿瘤^[1]^，也是中国发病率和死亡率最高的恶性肿瘤^[2]^。高危人群进行胸部低剂量计算机断层扫描(low-dose computed tomography, LDCT)筛查可以有效进行早期肺癌筛查，降低肺癌死亡率^[3]^。在中国，随着癌症早筛意识的提高，越来越多的人行LDCT进行肺癌筛查。肺部结节在临床中的检出也较为多见，文献^[4, 5]^报道对于肺结节的检出率可达21%-33%。对于发展中国家，尤其是结核高发地区，胸部CT发现的患者肺部结节的发病率可能更高^[6]^。

尽管有部分胸部CT筛查所见的肺部病灶是早期肺癌，但大多数的肺部结节是良性的^[7]^。对于肺部结节/占位的干预措施^[8]^，恶性倾向的可进一步行正电子发射计算机断层显像(positron emission tomography, PET)评估，但是PET诊断的敏感性和特异性也受病灶大小、病变性质等多种因素的影响^[8-11]^，最终需手术明确病理诊断并达到治疗效果，CT引导下穿刺并不作为常规推荐。良性倾向的肺结节可进行随访观察。对于中间界定属性的肺部病变^[8]^，可行CT引导下穿刺、气管镜下活检等有创检查辅助诊断，但部分肺部病灶因病变大小、位置等因素，无法完成活检而明确术前病理诊断。然而许多患者由于对于肿瘤的恐慌，对于性质中介(恶性风险3%-68%)的肺部病灶甚至低风险结节，更多地选择进行手术的干预。目前电视辅助胸腔镜手术(video-assisted thoracoscopic surgery, VATS)技术已经广泛推广。与传统开胸手术比较，VATS的围手术期风险小，术后恢复快，这也使得VATS成为针对肺部结节的重要诊疗手段。但是VATS仍有其一定的风险，包括各种围手术期并发症，而且肺部手术会降低患者肺功能。对于肺部良性病变进行手术治疗，给患者、家庭、社会造成经济负担，而且也带来了医疗资源的占用。良性肺部病灶形成的原因包括^[12, 13]^：慢性感染(结核、真菌、隐球菌、其他少见感染等)、炎性结节、错构瘤^[14]^、肺内淋巴结等，需要仔细进行鉴别。如何有效的对肺部病灶进行术前鉴别，如何降低可以避免手术切除的一些良性疾病的手术切除比例，是需要关注的问题。

本研究总结分析了我院胸外科及呼吸与危重症医学科接诊的术前考虑恶性肿瘤或恶性肿瘤不能除外，但手术病理为良性疾病的病例，以之提供临床借鉴。

## 1 资料与方法

### 1.1 研究对象

入选标准：①2017年1月1日-2018年12月31日，我院胸外科病房完成行肺部病灶手术切除患者; ②术前临床医生考虑肺内病灶为恶性肿瘤或恶性肿瘤不除外; ③经手术病理证实为良性病变; ④临床资料完善。排除标准：①术前临床医生评估为良性病变; ②术后病理发现癌前病变或恶性病变; ③以肺病灶活检为目的行肺部手术; ④术前进行气管镜检查、病灶穿刺活检、锁骨上淋巴活检、纵隔镜淋巴结活检等已明确疾病性质; ⑤其他(纵隔、食管、膈肌等)手术患者。

经我院临床病案室检索，2017年1月1日-2018年12月31日，我院胸外科完成肺部病灶楔形切除术、肺叶切除术或肺段切除术，且出院诊断为非恶性病变患者共计452例，其中125例患者于术前诊断肺隔离症、肺血管瘤、支气管扩张合并咯血、肺曲霉菌病、错构瘤、肺大疱等肺部良性病变，因原发疾病或疾病并发症住院行手术治疗。26例患者为出院后手术病理回报为不典型腺瘤样增生或原位腺癌，4例患者病理为其他恶性肿瘤。最终297例患者纳入本研究。该研究获得北京协和医院伦理委员会批准。

### 1.2 研究方法

回顾性收集患者病例资料，设计标准的研究性表格。收集临床信息包括：性别、年龄、住院时间、临床症状、病例随访时间、既往史、结核病史、吸烟史、肿瘤家族史、肺部病变个数、肺部病变性质、PET检查病变的最大标准摄取值(maximum standard uptake value, SUV_max_)、PET恶性倾向、手术方式、手术切除病灶个数、围手术期并发症、术中冰冻病理、手术病理和最终诊断。一个放射科医师、两个临床医生独立对肺内病灶进行评估，对于评估不一致经讨论后达成一致意见。如无本院影像学记录，则按照外院CT报告描述结果进行评估，如未提及，则记为未知。

### 1.3 评判标准

患者肺部病变影像学表现以手术前最后一次CT或PET所示的病灶大小、个数和影像学特征为准进行记录。对比手术前最后一次CT及初次发现肺部结节影像学检查，描述患者肺部结节变化。患者最终诊断依据手术病理结果，以病理科医师病理诊断报告为准。手术并发症记录手术后30 d内，出现手术相关的不良事件。

### 1.4 统计学方法

应用SPSS 17.0统计软件进行统计学分析并进行统计学绘图。计数资料采用率(%)表示，组间比较采用χ^2^检验; 计量资料采用均数±标准差(Mean±SD)表示，组间比较采用*t*检验，以*P* < 0.05为差异有统计学意义。

## 2 结果

### 2.1 基本临床特征

本研究共纳入297例肺部良性病变患者。同期就诊我院胸外科病房因肺部病灶行肺部手术治疗共计3, 033例，术前恶性不除外的肺部良性病变患者占比为9.8%。

患者的男女比例为158:139，平均年龄为(53.4±12.0)岁。100例(33.7%)患者有吸烟史; 吸烟患者中，平均吸烟量为(33.9±24.9)包年。8例(2.69%)患者有结核病史、23例(7.4%)有恶性肿瘤史、56例(18.9%)有肿瘤家族史，详见表 1。

**1:** 术前恶性不除外的肺部良性病灶的患者临床特征 Clinical characteristics of the patients with benign pulmonary lesions in which malignancy could not be excluded in preoperative assessment

Variable	Data
Age (yr)	53.4±12.0
Gender Male: Female	158:139
Smoking history	100 (33.7%)
Pasting medical history	163 (54.9%)
Respiratory diseases	8 (2.69%)
Tuberculosis	8 (2.69%)
Hypertension	75(25.3%)
Diabetes	36 (12.1%)
Coronary heart disease	14 (4.7%)
Malignant tumor	23 (7.7%)
Autoimmune disease	10 (3.4%)
Family history of cancer	56 (18.8%)
Lung cancer	26 (8.7%)
Non-lung cancer	30 (10.1%)
Without family history of cancer	241 (81.1%)

197例(66.3%)患者因体检行LDCT筛查发现肺部结节/占位，其中191例(64.3%)患者无症状。患者就诊的临床症状包括：咳嗽63例(21.2%)，咳痰30例(10.1%)，咯血/痰中带血19例(6.4%)，胸背痛20例(6.7%)，胸闷13例(4.4%)，发热8例(2.7%)，乏力1例(0.3%)，体重下降1例(0.3%)。

165例(55.6%)在发现肺内结节后在3月内行手术治疗，132例(44.4%)超过3个月的随诊，其中58例(19.5%)患者病灶有增大。

### 2.2 肺内病灶影像学

201例(67.7%)患者为单发结节，35例(11.8%)2个结节，12例(4.0%)3个结节，47例(15.8%)多发结节。肺部结节CT评估情况：可评估的323个病灶，最长径为(17.9±12.1)mm，影像学特征见表 2及图 1。

**2:** 术前恶性不除外、手术病理确诊肺部良性病灶的CT影像学特征 Chest CT features of surgical pathology confirmed benign pulmonary lesions in which malignancy could not be excluded in preoperative assessment

Variable	Data
Total number of evaluable nodules	323
Location	
Right upper lobe	91 (28.2%)
Right middle lobe	39 (12.1%)
Right lower lobe	67 (20.7%)
Left upper lobe	70 (21.7%)
Left lower lobe	56 (17.3%)
Peripheral type	241 (74.6%)
Central type	17 (5.3%)
Unknown	65 (20.1%)
Diameter of pulmonary lesions (cm)	
< 8	29 (9.0%)
8-20	205 (63.5%)
21-30	57 (17.6%)
> 30	32 (9.9%)
Attenuation	
Solid nodule	212 (65.6%)
Pure ground-glass nodule	46 (14.2%)
Part-solid nodule	33 (10.2%)
Unknown	32 (9.9%)
Spicule sign	
No	117 (36.2%)
Yes	71 (22.0%)
Unknown	135 (41.8%)
Lobulation	
No	109 (33.7%)
Yes	94 (29.1%)
Unknown	120 (35.2%)
Pleural indentation	
No	147 (45.5%)
Yes	81 (25.1%)
Unknown	95 (29.4%)
Vascular convergence sign	
No	94 (29.1%)
Yes	130 (40.2%)
Unknown	99 (30.7%)
Vacuole sign	
No	180 (55.7%)
Yes	23 (7.1%)
Unknown	120 (37.2%)
Satellite lesions	
No	177 (54.8%)
Yes	25 (7.7%)
Unknown	121 (37.5%)
Peri-lesion effusion	
No	246 (76.2%)
Yes	2 (0.6%)
Unknown	75 (23.2%)
CT: computed tomography.	

**1:**
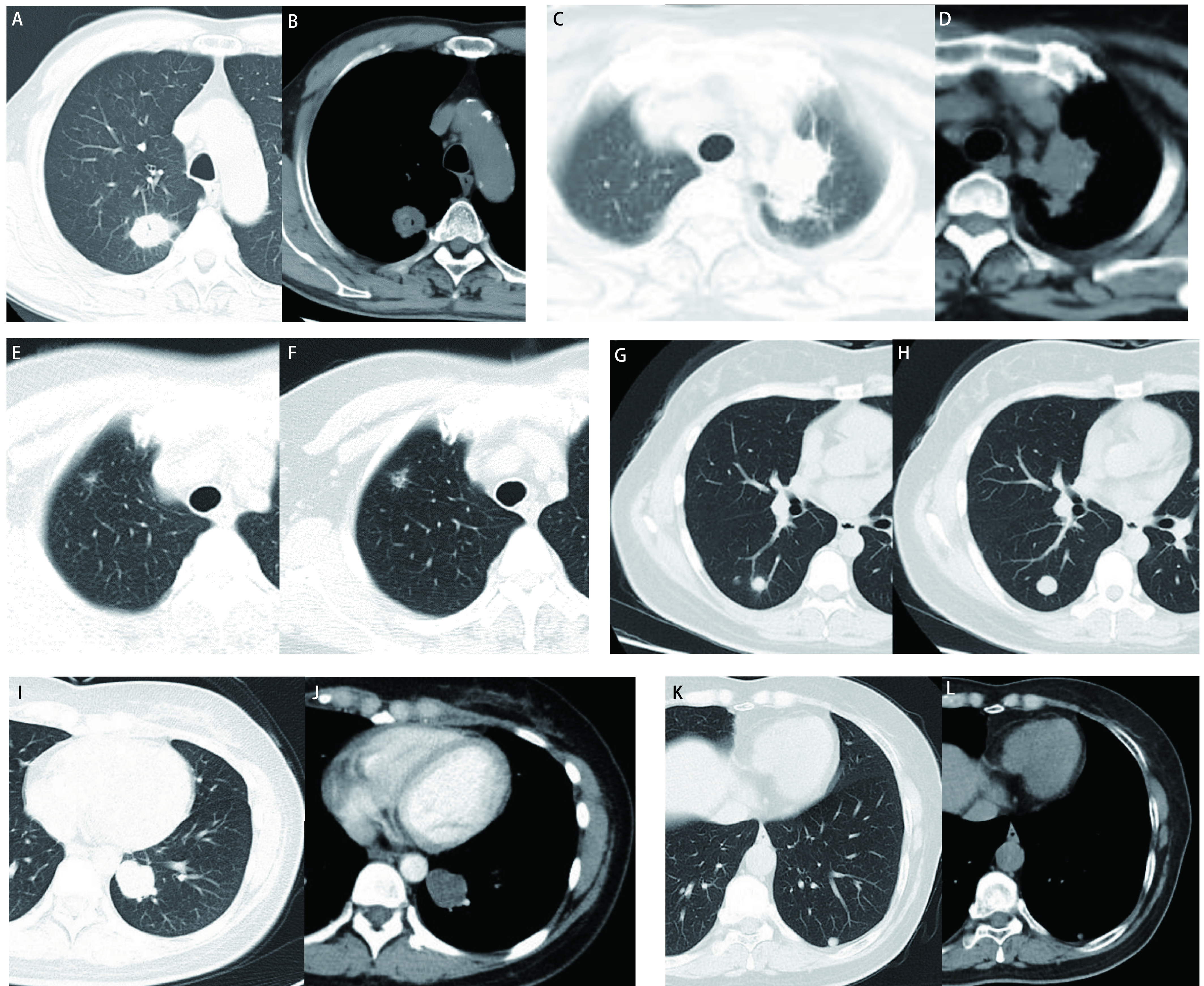
术前恶性不除外、手术病理确诊肺部良性病灶的CT影像学表现。A、B：右上肺结节伴有空泡征、毛刺和胸膜牵拉，病理结果：上皮样肉芽肿伴坏死，结核可能性大; C、D：左上肺占位，伴有点状钙化，病理为：上皮样肉芽肿结节伴多核巨细胞反应，抗酸染色(+)，病变符合结核; 右上肺部分实性结节(E)，随诊两年病灶有增大(F)，病理提示炎性结节; 右下肺结节(G)，随诊半年病灶有增大(H)，病理提示上皮样细胞伴多核巨细胞反应，考虑特殊感染; I、J：左下肺结节伴有分叶和血管挤压，病理提示错构瘤; 左下肺结节，随诊14个月病灶较前有增大(K、L)，病理提示肺内淋巴结。 Chest CT imaging findings of benign pulmonary lesions in which malignancy could not be excluded in preoperative assessment. A, B: Pulmonary solid nodule in the right upper lobe accompanied by vacuole sign, spicule sign and pleural indentation. Surgical pathology showed epithelioid granuloma accompanied by necrosis, with high probability of tuberculosis; C, D: Pulmonary mass in the left upper lobe accompanied by punctate calcification. Surgical pathology showed epithelioid granulomatous nodules with multinucleated giant cells and a positive acid-fast staining, confirming the diagnosis of tuberculosis. The solid nodule (E) in the right upper lobe increased after two years of follow-up (F), and surgical pathology suggested inflammatory nodule. The nodule in the right lower lobe (G) increased after six months of follow-up (H), and surgical pathology showed epithelioid cells with multinucleated giant cells, indicating chronic infection; I, J: Nodule in the left lower lobe accompanied by lobulation and vascular compression. Surgical pathology confirmed hamartoma; K, L: The diameter of nodule in the left lower lobe increased in 14 months follow-up LDCT. Surgical pathology diagnosis was intrapulmonary lymph node.

269例(90.6%)切除单个结节，22例(7.4%)切除2个结节，6例(2.0%)切除3个结节。

138例(46.5%)患者接受了PET/CT检查，108例(108/138, 78.3%)PET倾向恶性，24例(24/138, 17.4%)倾向良性，6例(6/138, 4.3%)无倾向性。PET SUVmax为3.72(范围：0-17.9)。

### 2.3 手术情况

292例(98.3%)行VATS胸腔镜手术，5例(1.7%)VATS转开胸手术。232例(78.1%)患者行肺楔形切除术，13例(4.4%)行肺段切除术，51例(17.2%)患者行肺叶切除术。4例(1.3%)患者出现手术并发症，分别为：脑梗1例，胸腔出血2例，严重气胸1例，经过治疗后均好转，无围手术期死亡。

### 2.4 病理

287例(96.6%)患者行术中冰冻病理检查，10例(3.4%)患者未送检冰冻病理。在送检病理的287例患者中，仅有1例术中病理见到肺泡上皮呈不典型增生，术后病理提示机化性肺炎，冰冻病理与术后病理一致率为99.7%。

术后病理结果包括：感染性病灶(结核分枝杆菌、分枝杆菌、隐球菌、曲霉菌、组织胞浆菌感染、Whipple病)、炎性结节、错构瘤、肺内淋巴结、机化性肺炎等。6例有2个不同性质的结节，共303个不同的病理结果。详见图 2。

**2:**
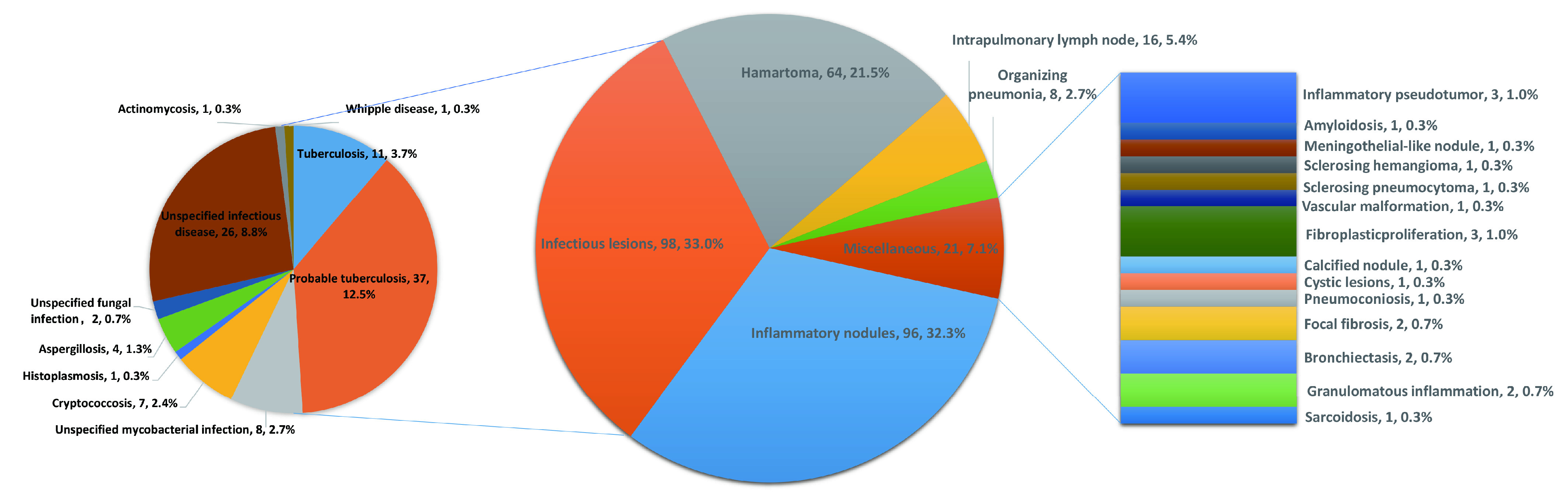
术前恶性病变不能除外的肺部良性病变手术病理结果 Surgical pathology results of benign pulmonary lesions which malignancy could not be excluded in preoperative assessment

## 3 讨论

本研究分析了我院2017年及2018年因不能除外恶性肿瘤的肺部病灶、最终行手术切除明确诊断的肺良性病灶的临床特征。研究发现：①297例患者纳入本研究，占我院肺部病灶行肺部手术治疗患者的9.8%，其中男性、不吸烟患者多见，多数患者行LDCT筛查发现肺部结节; ②行手术的肺内病灶8 mm-20 mm最为多见，实性为主，19.5%病灶随诊有增大，且PET/CT多数提示恶性可能或恶性不除外; ③292例患者(98.3%)行VATS治疗，82.5%患者行肺楔形切除术或肺段切除术，以最大程度的保护肺功能; ④病理以感染性病灶和炎性结节最为常见，错构瘤占第三位。

病理是肺部病变明确诊断的金标准，目前胸腔镜下的肺叶切除术或肺楔形切除术已经广泛推广，使得肺部病灶更易于通过手术明确病理。但是，由于LDCT的肺癌筛查项目的普及，使得发现肺内结节的人群数量明显增多，因此手术量也逐年上升。本研究发现，我院因不能除外恶性病变而行手术切除的肺部良性病变占比为9.8%。对于肺部良性病灶行手术切除，可以明确诊断，但是肺部手术应以肺楔形切除或肺段切除为主，以减少肺叶切除对于患者肺功能的影响。由于肿瘤位置及大小等原因，为了完整切除病灶，有17.7%患者行肺叶切除术，对于此类肺部病灶，更应该加强术前的判读评估。当然，对于较大的肺部良性病灶如曲霉菌球或有压迫症状的错构瘤，病灶切除也是一种治疗手段。

有多个模型进行预测肺内病变恶性的倾向性^[6, 11, 15, 16]^，目前也有多个人工智能的模型对于肺内的结节进行恶性倾向的预测，在临床实践中应用的时候，这种预测模型给临床医生一定的提示。从不同的筛查模型^[15, 16]^可以看出，病灶的大小仍是最直接的相关因素。与此对应，本研究中术前评估恶性不除外的323个肺部病灶中，≥8 mm的占91%，而 < 8 mm的结节仅为29个占9.0%，其中仅13例行手术切除。因此，对于超过8 mm的病灶，也需要进行相应的鉴别。此外实性结节相比纯磨玻璃密度结节及部分实性的结节，恶性可能相对少，因此对于实性结节的评估需更加谨慎^[15]^。结节的动态变化对于提示良恶性有重要的意义，若连续的影像学检查显示肺内病灶明显生长，则其恶性风险高，往往有必要进行组织取材诊断。但本研究中，有58例在术前观察期间病灶有增长。因此，病灶增长也并非是恶性所独有的特征，需要注意。对于持续稳定的病灶，则应尽量避免手术。保持稳定2年的实性结节和保持稳定5年的亚实性结节很可能为良性，可以避免立即行组织活检。此外，恶性病灶的影像学特征，例如分叶征、毛刺征、胸膜牵拉征等，在本研究的病例中也可见到。对于此类性质难以判断的病灶，在分析恶性病变相关影像学特征的时候，也需要注意有无特殊类型的钙化等代表良性病变的影像学特征。

为了降低良性肺部结节的手术率，需要更好的进行术前良恶性结节的判断。PET/CT对于恶性的敏感性和特异性分别为72%-94%和70%-95%^[9, 17, 18]^，但是不可否认的是，PET/CT存在假阳性^[18]^，多见于感染性的病灶，如真菌、结核，还可以见于非感染性的炎性结节。在本研究中，完善PET/CT的138例良性肺部病变患者，78.3%的患者PET/CT具有恶性倾向，因此PET/CT检查难以作为此类患者降低手术切除比例的措施。对于我院上述肺部病灶，感染性病灶仍为最多见，而炎性结节次之，随后是错构瘤和肺内淋巴结。对于上述感染性病变，可给予经验性抗感染治疗，但上述病变病原学以分枝感染为主，真菌次之，可能无法通过短程的抗感染方案进行消除。因此，怀疑感染性病灶可针对性的进行感染相关的检查，例如痰找结核杆菌、血隐球菌抗原等，进行鉴别，必要时进行气管镜等有创检查，明确病原及病理诊断。而本院病理良性结节中，有64例错构瘤及16例肺内淋巴结，上述病变在影像学上具有一定的特征性，因此，需要加强对上述病灶的影像学判断，以助于术前诊断。肺内淋巴结的CT的影像学特征，下肺多见，胸膜下(靠近胸膜1 cm以内)多见，多为小于12 mm，实性为主，可表现为椭圆形或三角形，边缘相对光滑，肺内淋巴结周围可见线样密度影^[19, 20]^，且淋巴结周围的线样密度影对于肺内淋巴结的诊断较为特异。此外，肺内淋巴结多与肺静脉相连，而极少与肺动脉相连，肺内淋巴结也有少部分会有增长的现象。错构瘤是肺内最常见的良性肿瘤^[14, 21]^，其CT影像学表现多为形体规则、边界清楚、部分患者可有爆米花样的钙化和脂肪密度^[22]^。但是部分不典型的错构瘤也难以与早期肺癌相鉴别^[23, 24]^，例如无明显钙化和脂肪密度的错构瘤病灶，也有部分错构瘤可有分叶及胸膜牵拉，部分随时间缓慢增长，因此需要更进一步的鉴别诊断。PET/CT对于错构瘤鉴别诊断有一定的价值，但是对于没有脂肪成分和典型钙化病灶的病灶，CT引导下的穿刺活检在一定程度上可以帮助做鉴别^[25]^。

术前肺部病变良恶性的判断，对于性质中介的结节(对于恶性风险在3%-68%的人群)，根据指南建议，进行非手术的活检可帮助鉴别良恶性病灶的性质^[8]^。目前非手术的活检的技术成熟，可考虑CT引导下经胸壁肺部病灶穿刺活检、支气管镜下经支气管针吸活检(transbronchial needle aspiration, TBNA)、经支气管超声引导鞘管引导下经支气管肺活检术(endobronchial ultrasound transbronchial lung biopsy using guide sheath, EBUS-GS-TBLB)。靠近胸壁的周围型结节或位置深但活检时不必穿过叶间裂且周围无大疱的病变，CT引导下的经胸壁活检是优选的活检手段，而且穿刺活检与手术病理的一致性很高。对于邻近支气管的病灶，支气管镜引导下的活检更为优选。电磁导航支气管镜可对CT获得的图像进行三维重建，创建支气管树的三维虚拟结构，并通过计算机准确定位，将探头引导至CT确定的病灶部位，可以提高外周病灶的病理检出率。对于临床判断恶性率低或者中介的肺部病灶，若患者意愿强烈，术前的病理活检评估有一定的价值。但是，CT引导下穿刺或者气管镜下的活检，也有其局限性，不是所有的肺部病灶(尤其是肺部小结节)都能进行术前的病理活检，因此，VATS也可以作为一种获取病理的诊断手段。

综上，本研究对于我院因不能除外恶性肿瘤而进行手术切除的肺部良性病灶进行了总结分析。我院手术切除的这类病灶，影像学表现以实性病灶多见，但多具有提示恶性的影像学特征。手术方式VATS最为常用，并行肺楔形切除或肺段切除最大程度的保护肺功能，术中进行冰冻病理明确诊断，围手术期并发症少。病理结果提示在这类病灶中，感染性疾病和炎性结节最为常见，错构瘤第三。为了更好地进行鉴别诊断，对于性质中介的肺结节，建议行术前活检，明确病原和病理，指导后续诊治，无法行术前活检的病灶，VATS也是一种明确病原病理的重要活检方式。

**Author contributions**

Liu YJ, Xu Y, Li SQ and Wang MZ conceived and designed the study. Liu YJ, Chen MJ, Guo C, Zhong W, Ye QY, Zhao J, Zhou Q, Gao XX, Liu XY, Liang HG, Shi YQ, Jiang DLN, Liu HS, Xu Y, Li SQ and Wang MZ contributed data collection. Liu YJ, Chen MJ, Guo C, Xu Y, Li SQ and Wang MZ provided critical inputs on design, analysis, and interpretation of the study. Liu YJ, Chen MJ, Guo C, Xu Y, Li SQ and Wang MZ contributed manuscript writing. All the authors had access to the data. All authors read and approved the final manuscript as submitted.
